# The Characteristics of TB Epidemic and TB/HIV Co-Infection Epidemic: A 2007–2013 Retrospective Study in Urumqi, Xinjiang Province, China

**DOI:** 10.1371/journal.pone.0164947

**Published:** 2016-10-21

**Authors:** Wang Wei, Zhang Wei-Sheng, Alayi Ahan, Yan Ci, Zhang Wei-Wen, Cao Ming-Qin

**Affiliations:** 1 Department Epidemiology and Health Statistics, School of Public Health, Xinjiang Medical University, Urumqi, Xinjiang, China; 2 Urumqi Center for Disease Control and Prevention (CDC), Urumqi, Xinjiang, China; Temple University School of Medicine, UNITED STATES

## Abstract

**Objective:**

This study was aimed to find out epidemiologic characteristic of tuberculosis (TB) cases, and Human Immunodeficiency Virus (HIV) positive cases among TB patients (TB/HIV co-infection) through demographic, temporal, and spatial study in Urumqi.

**Methods:**

Descriptive statistics and multivariate logistic regression were applied to identify the epidemiologic characteristics and risk factors of TB epidemic and TB/HIV co-infection epidemic. All addresses of each TB case, TB/HIV co-infection case, and administrative street were transformed into geographical coordinate. Subsequently, the geocoded address for 82 streets was transformed into a dot map used as the basis of spatial datasets. In addition, the paper also used quantile map and the spatial scan statistic in order to identify the spatial distribution and spatial clusters of TB epidemic and TB/HIV co-infection epidemic.

**Result:**

There was a declining trend of the notification rates of TB epidemic from 2007 to 2009, as well as a rising trend from 2010 to 2013. However, the notification rates of TB/HIV co-infection epidemic showed a rising trend from 2007 to 2010, and a declining trend from 2011 to 2013. Moreover, a significant share of TB epidemic and TB/HIV co-infection epidemic happened between the age of 15 to 45 years old, indicating an increase in risk of TB and TB/HIV infection. It is worth noting that the risk of HIV infection for male TB patients was 2.947 times (95% CI [2.178, 3.988]) than that of female patients. Han ethnicity and Uygur ethnicity in urban region accounted for a large proportion of total TB and TB/HIV co-infection cases. Most of the TB cases of minorities in Urumqi showed a statistically significant increase in risk of HIV infection than Han ethnicity in Urumqi. In addition, the spatial distribution of TB epidemic and TB/HIV co-infection epidemic was highly skewed. Most of the local clusters were located in urban area and rural-urban continuum where showed an increase in risk of TB and TB/HIV infection.

**Conclusion:**

The epidemiologic and spatial-temporal analysis of TB epidemic and TB/HIV co-infection epidemic demonstrates a potential connection between TB and HIV in Urumqi. Demographic, temporal, geographic factors are the reasons of causing TB and TB/HIV co-infection epidemic.

## Introduction

Tuberculosis (TB) is contagious and airborne, ranking as a leading cause of death worldwide alongside Human Immunodeficiency Virus (HIV) infection [1]. Despite being preventable and treatable, TB remains the most common life-threatening opportunistic infection and a leading cause of deaths among people living with HIV/AIDS (PLWHA) [2]. Meanwhile, with the increase of HIV positive TB patients and the decrease of treatment success rate among HIV positive TB patients [2], there is no positive trend for TB epidemic and TB/HIV co-infection epidemic, which has galvanized consensus over growing TB/HIV case.

As the report of the Chinese Center for Disease Control and Prevention (CDC) pointed, Xinjiang province has been confirmed as one of the high TB and HIV burdened provinces of China, according to data collected during the period from 2010 to 2013 [3]. Urumqi is the capital of Xinjiang, with a population of 2,493,519 in 14,200 km^2^, which accounted for a large proportion of total TB and HIV cases reported in Xinjiang. In 2011, TB prevalence in Urumqi reached up to 1,526.12/100,000 [4]. At the same time, HIV patients in Urumqi accounted 34.6% of total HIV cases reported in Xinjiang [4]. The infected ration of TB/HIV co-infection in Urumqi region is estimated at 142 cases per 1,000 TB patients from September 2007 to December 2009 [5]. In general, the epidemic of TB and TB/HIV co-infection is not optimistic in Urumqi in recent years.

Historically, special risk factors conduced to high prevalence of TB and HIV micro-epidemics in specific areas in Urumqi [6–8]. For example, there are 553,000 urban migrants, who are difficult to follow-up because they do not have stable living condition [9–11]. Besides, due to have a limited knowledge of Mandarin, minorities in multiethnic area usually have lower social and economic level [12], leading up to a limited knowledge of TB and HIV [13], and a higher risk of TB and HIV than Han [9]. Accordingly, despite Urumqi Center for Disease Control and Prevention (CDC) had adopted a series of initiatives to prevent and control the TB and HIV epidemics, the special groups in specific areas still have high prevalence of TB and HIV. Therefore, there is an urgent need to identify the risk factors of TB and TB/HIV co-infection in order to promote the development of regional prevention strategies in Urumqi.

In all infectious diseases research, it is important to evaluate whether observed cases are representative for general cases. Such spatial clustering analysis is an important implement for diseases spatial randomness tests. Furthermore, the spatial clustering tests would provide location information of local clusters and the risk of each cluster, used as the evidence of diseases prevention and control [14]. Thus, it is necessary to detect the spatial clusters of TB epidemic and TB/HIV co-infection epidemic, for the purpose of examining whether the TB and TB/HIV co-infection cases are randomized distributed or not, as well as the risk of TB and TB/HIV co-infection for each clustered street.

Therefore, the main objectives of this study were to: (1) use descriptive statistics and multivariate logistic regression to estimate the risk factors of TB epidemic and TB/HIV co-infection epidemic; (2) use GIS to explore the geospatial characteristic and examine data aggregated to the level of the street; (3) employ SaTScan tests to examine spatial clusters of TB epidemic and TB/HIV co-infection epidemic, along with the risk of TB and TB/HIV co-infection for each clustered street in Urumqi region.

## Methods

### Ethics Statement

The study protocol was approved by Urumqi Center for Disease Control and Prevention. This article does not contain any studies with human participants performed by any of the authors. All patient information was anonymized and de-identified prior to analysis. Therefore, no ethics approval was required by our Investigation Review Board.

### Data sources and materials

Surveillance information for TB and TB/HIV is collated through the tuberculosis surveillance center and the Tuberculosis Registration Systems. Besides, CDC supplies all the case of TB, HIV diagnoses, with all tests following the principle of voluntariness. In the data set, it has covered the number of cases concerning HIV positive among TB people reported from 2007–2013 for each street in Urumqi. Therefore, the population data from Statistical Yearbook are able to be publicly available.

### Statistical Analysis

Descriptive statistics were used to explore the demographic characteristics and temporal trends of TB epidemic and TB/HIV co-infection epidemic, which includes age, occupation, and ethnic group, etc. Multivariate Logistic Regression was performed to seek correlation between TB/HIV co-infection and risk factors such as gender (male or female), age, ethnic group, and living region (rural area or urban area). All p-values were two-tailed, and values less than 0.05 were considered statistically significant. The number of TB cases or TB/HIV co-infection cases was combined to examine spatial-temporal variations for each year and each street.

In order to explore spatial variations for TB and TB/HIV co-infection epidemics and build spatial datasets, the paper transformed the geographical location of TB and TB/HIV co-infection cases into geographical coordination at first. The geocoded address for each street was then transformed into a dot map, with each dot representing the center of gravity for each street. BaiDu API assists to supply the longitude and latitude for each street and each case. Finally, the number of TB and TB/HIV co-infection cases was counted at street-level, used for connecting with spatial analysis dataset. Besides, for describing the spatial distribution of TB epidemic and TB/HIV co-infection epidemic, this paper applied quantile map, which along with the spatial databases are established by Geoda 1.6.7.

Regarding the dimensions of space, the identification of the local clusters of TB epidemic and TB/HIV co-infection epidemic across different streets geographically was completed by SaTScan™ v9.4.2, a spatial scan statistic developed by Kulldorff [15]. The spatial scan statistic imposes a circular window on the dot map, allowing the centroid to move across 82 streets; during the process of analysis, it used the centroids of the “streets”. Then, the radius of the window is changed continuously to take any value between zero and some upper limit [16].

The likelihood is calculated for each circle, and the definition of the test statistic stands for the maximum likelihood over all circles evaluated. Let D_*(i*,*j)*_ be the total number of cases in street i and its closest street j. And let U_*(i*,*j)*_ be the population size of cases in street i and its closest street j. In mathematical notation:

$$T=\underset{i,j}{\min\frac{L_{\left(i,j\right)}}{L_{0}}}I\left(D_{\left(i,j\right)}>\frac{C}{N}U_{\left(i,j\right)}\right)$$

Where L_(*i*,*j*)_ refers to the likelihood under the alternative hypothesis that there is a cluster in location i and its closest neighbors, L_0_ represents the likelihood under the null hypothesis and It can be shown that:

$$\frac{L_{\left(i,j\right)}}{L_{0}}=\left(\frac{D_{\left(i,j\right)}}{U_{\left(i,j\right)}\frac{C}{N}}\right)D_{\left(i,j\right)}{\left(\frac{C-D_{\left(i,j\right)}}{N-U_{\left(i,j\right)}\frac{C}{N}}\right)}^{C-D_{\left(i,j\right)}}$$

*I()* stands for the indicator function, I = 1 when $D_{\left(i,j\right)}>\frac{C}{N}U_{\left(i,j\right)}$, otherwise *I* = 0. The null hypothesis of no clustering is rejected when T is large.

## Results

### The temporal and demographic characteristic of TB and TB/HIV co-infection in Urumqi region, 2007–2013

**Fig 1:**
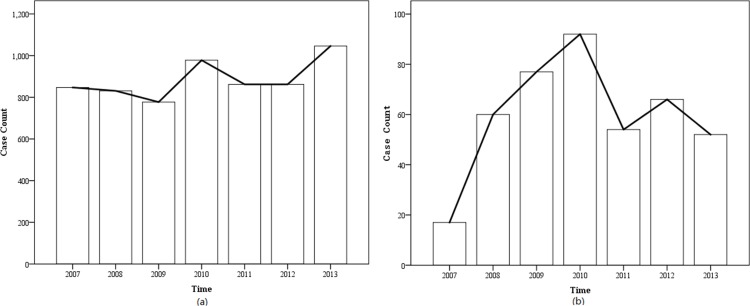
Temporal trends in TB epidemic and TB/HIV epidemic, Urumqi, 2007 to 2013: (a) Temporal trends of TB epidemic; and (b) Temporal trends of TB/HIV co-infection epidemic.

A total of 6,203 TB patients in Urumqi region from 2007 to 2013 were taken as cases. 2,836 TB cases accepted HIV tests, with 418 cases being HIV positive, and 2,418 cases being HIV negative. Fig 1 showed the temporal trends in TB and TB/HIV co-infection epidemics, in Urumqi, from 2007 to 2013. The notification rates of TB epidemic in the region appeared to decrease from 36.62 in 2007 to 32.22 per 100,000 people in 2009, but increase from 34.56 in 2011 to 39.78 per 100,000 people in 2013. Furthermore, the notification rates of TB/HIV co-infection epidemic in the region tended to increase from 0.73 in 2007 to 3.75 per 100,000 people in 2010, but decrease from 3.75 in 2010 to 1.98 per 100,000 people in 2013.

Baseline demographic characteristics of TB stratified by time categories are presented in Table 1. The number of male cases was twice than that of female cases in any given year. A significant share of TB infections happened among adults between the age of 15 to 30 years old (around 31.6%) and 30 to 45 years old (around 29.1%) respectively. TB infections occurred most frequently among Han (around 56.3%), followed by Uygur (around 30.5%), and Hui (around 10.3%). Meanwhile, urban region accounted for 76.1% of total TB cases, and rural region accounted for 23.9%. Table 2 showed demographic characteristics of TB/HIV co-infection cases in Urumqi from 2007 to 2013. It can be seen that there is a 4.9:1 ratio for the male-to-female of HIV positive outcomes based on TB infectious rate. The 418 TB/HIV co-infection cases were composed of 335(80.1%) Uygur, 54(12.9%) Han and 23(5.5%) Hui. 311 cases were aged 30–45 years old, 72 (18.2)15-30 years old and 31(7.4%) 45–60 years old. The number of rural region cases (13.2%) was always less than that of urban region cases (86.8%) in any given year.

**Table 1:** The demographic characteristics of TB cases in Urumqi from 2007 to 2013(N, %).

characteristics		2007	2008	2009	2010	2011	2012	2013
**Gender**	Male	**553 (65.3)**	**541(65.1)**	**547(70.4)**	**639(65.3)**	**526(61.0)**	**540(62.6)**	**639(61.1)**
	Female	294(34.7)	290(34.9)	230(29.6)	339(34.7)	336(39.0)	322(37.4)	407(38.9)
**Ethnic group**	Han	**827(97.6)**	**203(24.4)**	**410(52.8)**	**498(50.9)**	**458(53.1)**	**495(57.4)**	**602(57.6)**
	Uygur	**15(1.8)**	**585(70.4)**	**235 (30.2)**	**295(30.2)**	**226(26.2)**	**242(28.1)**	**295(28.2)**
	Hui	1(0.1)	33(4.0)	101(13.0)	140(14.3)	153(17.7)	98(11.4)	111(10.6)
	Kazakh	4(0.5)	8(1.0)	25(3.2)	35(3.6)	16(1.9)	19(2.2)	26(2.5)
	Other	0(0.0)	2(0.2)	6(5.9)	10(1.0)	9(1.0)	8(0.9)	12(1.0)
**Age (years)**	0–15	9(1.1)	10(1.2)	4(0.5)	15(1.5)	9(1.0)	8(0.9)	1(1.1)
	15–30	**323(38.1)**	**318(38.3)**	**287(36.9)**	**307(31.4)**	**245(28.4)**	**225(26.1)**	**255(24.4)**
	30–45	**249(29.4)**	**250(30.1)**	**226(29.1)**	**279(28.5)**	**249(28.9)**	**252(29.2)**	**298(28.5)**
	45–60	100(11.8)	90(10.8)	95(12.2)	137(14.0)	134(15.5)	163(18.9)	167(16.0)
	>60	166(19.6)	163(19.6)	165(21.2)	240(24.5)	225(26.1)	214(24.8)	315(30.1)
**Region**	Urban	**675(79.7)**	**659(79.3)**	**491(63.2)**	**686(70.1)**	**583(67.6)**	**595(69.0)**	**787(75.2)**
	Rural	172(20.3)	172(20.7)	286(36.8)	292(29.9)	279(32.4)	267(31.0)	259(24.8)

**Table 2:** The demographic characteristics of TB/HIV cases in Urumqi from 2007 to 2013(N, %).

characteristics		2007	2008	2009	2010	2011	2012	2013
Gender	Male	**15(88.2)**	**51(85.0)**	**64(83.1)**	**78(84.8)**	**47(87.0)**	**56(84.8)**	**36(69.2)**
	Female	2(11.8)	9(15.0)	13(16.9)	14(15.2)	7(13.0)	10(15.2)	16(30.8)
Ethnic group	Han	**15(88.2)**	**3(5.0)**	**8(10.4)**	**9(9.8)**	**3(5.6)**	**5(7.6)**	**11(21.2)**
	Uygur	**2(11.8)**	**53(88.3)**	**64(83.1)**	**76(82.6)**	**47(87.0)**	**56(84.8)**	**37(71.2)**
	Hui	0(0.0)	2(3.3)	4(5.2)	6(6.5)	4(7.4)	4(6.1)	3(5.8)
Age (years)	15–30	**7(41.2)**	**15(25.0)**	**25(32.5)**	**12(13.0)**	**6(11.1)**	**3(4.5)**	**4(7.7)**
	30–45	**10(58.8)**	**43(71.7)**	**45(58.4)**	**75(81.5)**	**41(75.9)**	**52(78.8)**	**45(86.5)**
	45–60	0(0.0)	2(3.3)	5(6.5)	5(5.4)	7(13.0)	10(15.2)	2(3.8)
	>60	0(0.0)	0(0.0)	2(2.6)	0(0.0)	0(0.0)	1(1.5)	1(1.9)
Region	Urban	**10(58.8)**	**49(81.7)**	**64(83.1)**	**85(92.4)**	**46(85.2)**	**61(92.4)**	**48(92.3)**
	Rural	7(41.2)	11(18.3)	13(16.9)	7(7.6)	8(14.8)	5(7.6)	4(7.7)

A multivariate logistical regression model is shown in Table 3. According to the influential factors analysis for HIV positive among TB patients, it turned out that the risk of HIV infection for male cases was 2.947 times than that in female cases. Statistically, there was a significant increase in risk of HIV infection for Uygur ethnicity (OR = 9.578, 95% CI [7.085, 13.440]), Hui ethnicity (OR = 2.794, 95% CI [1.625, 4.805]), and other ethnicities (OR = 3.578, 95% CI [1.134, 11.288]) in Urumqi. Patient with age between 0–30 years old (OR = 0.193, 95% CI [0.143, 0.260]), 45–60 years old (OR = 0.157, 95% CI [0.104, 0.236]), and ≥ 60 years old (OR = 0.019, 95% CI [0.007, 0.052]) were all statistically significant for lower risk of HIV infection in Urumqi. Although it was not significant at the threshold p-value of 0.05, urban cases (OR = 1.244, 95% CI [0.871, 1.777]) might share a higher risk in HIV infection, but Kazakh ethnicity might share a lower risk in HIV infection (OR = 0.546, 95% CI [0.127, 2.351]).

**Table 3:** The characteristics and risk factors for Human Immunodeficiency Virus (HIV) infection among Tuberculosis (TB) patients who underwent HIV testing (n = 2, 836).

Characteristic		Tested	HIV infection		P-value	OR (95% CI)
			n	%		
Gender	Male	1818	347	83.0	<0.001	2.947(2.178, 3.988)
	Female	1018	71	17.0	-	1.00
Ethnic group	Han	1404	54	12.9	-	1.00
	Uygur	1120	335	80.1	<0.001	9.758 (7.085, 13.440)
	Hui	220	23	5.5	<0.001	2.794 (1.625, 4.805)
	Kazakh	59	2	0.5	0.416	0.546 (0.127, 2.351)
	Other	33	4	1.0	0.030	3.578(1.134, 11.288)
Age (years)	0–30	826	72	17.2	<0.001	0.193(0.143, 0.260)
	30–45	918	311	74.4	-	1.00
	45–60	465	31	7.4	<0.001	0.157(0.104, 0.236)
	>60	627	4	1.0	<0.001	0.019(0.007, 0.052)
Region	Urban	2344	363	86.8	0.230	1.244(0.871, 1.777)
	Rural	492	55	13.2	-	1.00

### Spatial patterns of TB and TB/HIV co-infection epidemics

**Fig 2:**
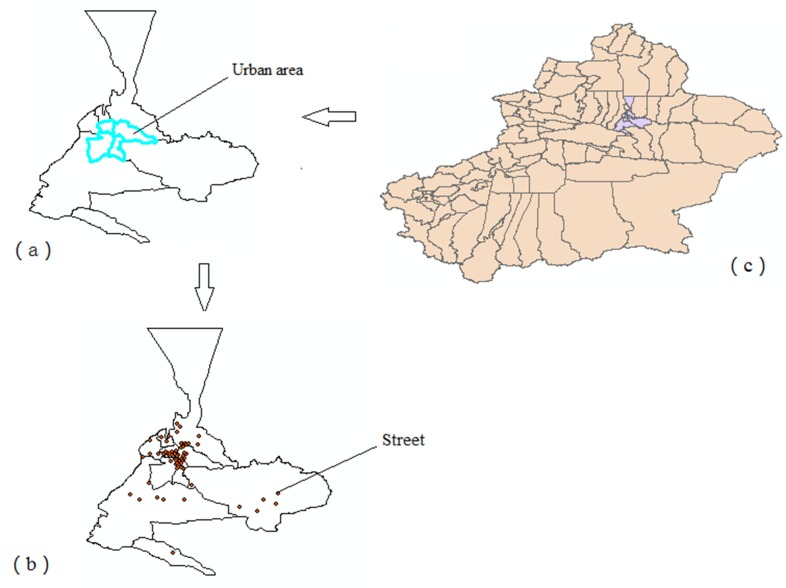
Overview of the study area, two different spatial scale are shown: (a) 8 counties; (b) 82 administrative streets (red dots) in Urumqi region; (c) location of the study area in Xinjiang province, China.

**Fig 3:**
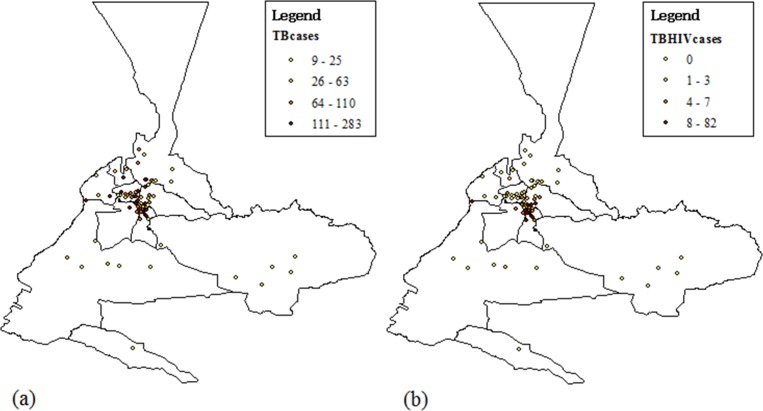
Quantile map of TB epidemic and TB/HIV co-infection epidemic: (a) Quantile map of TB epidemic; (b) Quantile map of TB/HIV co-infection epidemic.

Fig 2 showed the administrative map of Urumqi region in Xinjiang provinces, China, in which the spatial distribution of TB epidemic and TB/HIV co-infection epidemic could be seen through quantile map (Fig 3). The spatial distribution of TB is highly skewed, ranging from a minimum of 9 cases to a maximum of 283 cases. The mean number of cases per street is 45.00 with a standard deviation of 61.79, and the median is 1 case per street with an interquartile range from 20.00 to 107.25. Similar to the spatial distribution of TB, the spatial distribution of TB/HIV co-infection epidemic is also highly skewed, ranging from a minimum of 0 case to a maximum of 82 cases. The mean number of cases per street is 5.16 with a standard deviation of 11.44, and the median is 1 case per street with an interquartile range from 0 to 6.00. These descriptive statistics indicate that TB epidemic and TB/HIV co-infection epidemic spread out across most streets and exist geographic dispersion in Urumqi.

**Fig 4:**
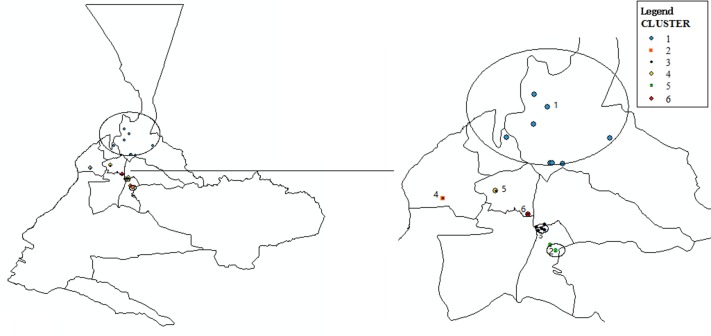
Spatial clustering map of TB epidemic with the method of Kulldorff during 2007–2013: Cluster #1: 555 observed cases and 584 expected (RR = 0.95, LLR = 47.59, P = 0.001). Cluster #2: 54 observed cases and 7 expected (RR = 8.30, LLR = 19.87, P = 0.001). Cluster #3: 361 observed cases and 477 expected (RR = 0.74, LLR = 11.69, P = 0.001). Cluster #4: 160 observed cases and 129 expected (RR = 1.25, LLR = 10.99, P = 0.001). Cluster #5: 144 observed cases and 56 expected (RR = 2.60, LLR = 9.38, P = 0.005). Cluster #6: 241 observed cases and 163 expected (RR = 1.50, LLR = 2.55, P = 0.918). Clusters #1, #3, and #5 are the spatial clusters of TB/HIV co-infection epidemic.

The clustered analysis of TB epidemic was detected yearly from 2007 to 2013. The clustered analysis with the spatial scan statistics of Kulldorff evidenced 6 spatial clusters (Fig 4). The first cluster, with centroid on street No.1 (radius 16.63 km), contained 555 observed TB cases to be compared with 584 expected TB cases (Relative Risk = 0.95, Log likelihood ratio = 47.59, P = 0.001). The second most likely cluster had centroid on street No.4 (radius 0 km), with 54 observed TB cases for 7 expected TB cases (Relative Risk = 8.30, Log likelihood ratio = 19.87, P = 0.001). The third most likely cluster was observed on street No.3 (radius 1.29 km) and contained 361 TB cases (Relative Risk = 0.74, Log likelihood ratio = 11.69, P = 0.001). Finally, the rest of spatial clusters appeared in street No.5 (radius 0 km), street No.2 (radius 1.87 km), and street No.6 (radius 0 km).

**Fig 5:**
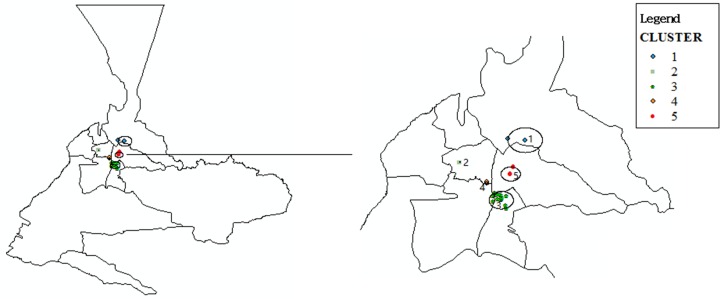
Spatial clustering map of TB/HIV co-infection epidemic with the method of Kulldorff during 2007–2013: Cluster #1: 2 observed cases and 28 expected (RR = 0.07, LLR = 22.27, P = 0.001). Cluster #2: 4 observed cases and 13 expected (RR = 0.29, LLR = 4.76, P = 0.155). Cluster #3: 162 observed cases and 131 expected (RR = 1.39, LLR = 3.24, P = 0.500). Cluster #4: 6 observed cases and 29 expected (RR = 0.20, LLR = 2.83, P = 0.635). Cluster #5: 15 observed cases and 8 expected (RR = 1.81, LLR = 1.98, P = 0.884). Clusters #1, #2, and #3 are the spatial clusters of TB epidemic.

The clustered analysis of TB/HIV co-infection with the spatial scan statistics evidenced 5 spatial clusters (Fig 5). The first cluster, with centroid on street No.1(radius 4.21 km), contained 2 observed TB/HIV co-infection cases and 28 expected TB/HIV co-infection (Relative Risk = 0.07, Log likelihood ratio = 22.27, P = 0.001). Although there was no significant threshold with p-value of 0.05, the other 4 spatial clusters were detected with the spatial scan statistics (p-values of 0.155, 0.500, 0.635, and 0.884). They had centroid on street No.2 (radius 0 km), street No.3 (radius 2.98 km), street No.4 (radius 0.44 km), and street No.5 (radius 2.38 km).

## Discussion

The observed temporal trends of TB epidemic kept declining from 2007 to 2009, and started to rise since 2010. This may be because the observed incidence rate of smear-positive demonstrated a relatively stable trend from 2007 to 2009, and a rising trend since 2010 [4, 17]. Compared with the observed temporal trends of TB, the observed temporal trends of TB/HIV co-infection epidemic varied markedly across all period, for example, in 2010, there had an absolute downward trend in TB/HIV co-infection case count. Subsequently, the similar trend had been reported by Peierdun MIJITI [18], which pointed out that the trend was caused by newly-diagnosed HIV/AIDS cases. Although all results reflected that the TB/HIV co-infection rate (14.7%) from 2007 to 2013 in this study was higher than those reported in China [4, 8], it was consistent with the finding of CHEN Yang-gui that the TB/HIV co-infection rate in Urumqi was 14.1% in 2007–2009 [5]. Therefore, the screening of HIV/AIDS patients for TB is of great importance for effective control of TB.

Compared with female TB cases, the number of male cases is much bigger, and indicates a higher risk in HIV infection. There are more TB or TB/HIV co-infection cases observed in younger population (< 45 years old). Besides, it turned out that TB patients aged 30–45 had higher risk of HIV infection than other age groups, which is in accord with the conclusion of Peierdun MIJITI [18], Li WG [4], and Zhang Y [8]. This may be because, compared with other age group, people in 30–45 years would be sexually active, increasing the chances of an individual’s risky sexual behavior [8]. In addition, Han and Uighur accounted for a large proportion in total number of TB and TB/HIV co-infection cases in Urumqi. Compared with other minorities, a large proportion of Uighur ethnicity suffered from TB and TB/HIV co-infection, due to using drug [8], unfavorable socioeconomic conditions [17], ethnic customs [18], and other risk factors associated with TB and HIV prevalence. This may be the reason that Uygur ethnicity cases accounted for a large proportion of the total number of TB/HIV co-infection cases, and shared a higher risk in TB/HIV co-infection in Xinjiang.

The visualization of the TB epidemic and TB/HIV co-infection epidemic in Urumqi over a 7-year period showed that the study districts are geographically clustering. Meanwhile, according to the spatial analysis results, it indicated that TB epidemic and TB/HIV co-infection epidemic shared almost same spatial clusters during the period from 2007 to 2013. Socio-economic status of neighborhoods is associated with geographic clustering of TB and HIV related outcomes [19–22]. The paper also evidenced that socioeconomic status and population density, urban migrants would be the important neighborhood characteristics associated with the spatial distribution of TB epidemic and TB/HIV co-infection epidemic in Urumqi. There would have higher risk of TB/HIV co-infection for streets in urban area with higher socioeconomic status, greater population density, and more urban migrants. In addition, for streets with greater population density and more urban migrant, it had higher risk of TB infection.

All results indicated that there is a close correlation between TB epidemic and HIV epidemic. Several studies have proved that HIV epidemic is strongly associated with TB at the early stages, which has become one key factor undermining global TB control [23–24]. In this research, there are similar demographic characteristics and spatial-temporal trends between TB epidemic and TB/HIV co-infection epidemic, evidencing the potential associations between TB epidemic and TB/HIV co-infection epidemic in Urumqi.

However, there are several limitations deserved to be discussed. Firstly, the data was extracted from the tuberculosis surveillance center and the Tuberculosis Registration Systems which failed to cover HIV/TB surveillance data. Secondly, there are several clustering analysis methods, and the choice of the methods might affect the result of disease clustering analysis [15]. Although the spatial scan statistics is the most commonly used disease clustering method, the result may be limited because only one method was applied in this research. Thus, further studies should utilize many different tests to study spatial clusters for TB or TB/HIV co-infection epidemic.

## Conclusions

In general, this paper explored the epidemiologic characteristic and the corresponding risk of TB and TB/HIV co-infection epidemics, which can provide an evidence for further socio-demographic predicting research on the TB and TB/HIV co-infection in Urumqi. Considering that the similar demographic characteristics as well as clustered districts of TB epidemic and TB/HIV co-infection epidemic have been observed, the related government organizations should carry out a wide-spread HIV screening on TB patients in Urumqi, for the purpose of improving the discovery rate of HIV on TB patients, providing in time prevention services for people with TB/HIV co-infection, and effectively reducing the impact of HIV on population health.
